# Biological implications of PTEN upregulation and altered sodium/iodide symporter intracellular distribution in resveratrol-suppressed anaplastic thyroid cancer cells

**DOI:** 10.7150/jca.48180

**Published:** 2020-10-04

**Authors:** Le Xiong, Jun-Hua Nie, Xiao-Min Lin, Jian-Bin Wu, Zhen Chen, Bo Xu, Jia Liu

**Affiliations:** 1 South China University of Technology School of Medicine, Guangzhou 510006, China.; 2 Department of Thyroid Surgery, Guangzhou First People's Hospital, South China University of Technology School of Medicine, Guangzhou 510180, China.; 3 Department of Oncology, First Affiliated Hospital, Guangzhou University of Chinese Medicine, Guangzhou 510405, P.R. China.

**Keywords:** PTEN, NIS, intracellular distribution, resveratrol, anaplastic thyroid cancer

## Abstract

**Objective:** Anaplastic thyroid cancer/ATC is a highly aggressive malignancy with extremely poor prognosis. Resveratrol/Res promotes re-differentiation of cancer cells and exerts inhibitory effects on ATC cells. Sodium/iodide symporter/NIS and phosphate and tension homology deleted on chromsome ten/PTEN levels are positively correlated with the grade of thyroid cancer differentiation, while the impact of Res on them remain unknown.

**Materials and Methods:** The patterns of NIS and PTEN expression and intracellular distribution in THJ-16T and THJ-21T ATC and Nthy-ori 3-1 normal thyroid cells and their relevance with Res-caused ATC suppression were investigated via multiple experimental methods. E-cadherin was cited as a re-differentiation biomarker of ATC cells.

**Results:** MTT and EdU cell proliferation assays showed distinct growth suppression in ATC cells after Res treatment. TUNEL staining revealed extensive apoptosis of Res-treated THJ-16T and THJ-21T rather than Nthy-ori 3-1 cells. Western blotting, immunocytochemical/ICC and double-labeled immunofluorescent/IF staining showed increased PTEN levels accompanied with distinct NIS and PTEN nuclear co-translocation in Res-treated THJ-16T and THJ-21T cells. E-cadherin but not NIS appeared on the outer membrane.

**Conclusion:** PTEN upregulation and the concurrent NIS and PTEN nuclear translocation in Res-suppressed ATC cells may indicate the better therapeutic outcome and would be a group of beneficial prognostic factors of ATCs.

## Introduction

The anaplastic thyroid cancer (ATC) has extremely poor prognosis, because over 70% of patients have distant metastasis at the time of diagnosis 1. Several remedies have been available to treat ATCs, of which systemic chemotherapy combined with radioiodine therapy is the optimal choice 2. Nevertheless, the therapeutic outcome is not promising because of the undifferentiated state and sodium/iodide symporter (NIS) reduction of ATCs 3. NIS, a SLC5A5 encoded transmembrane glycoprotein, is located on the basement membrane of thyroid follicular cells and mediates the entry of iodine into cells and participate in the synthesis of thyroid hormones 4. The correlation of NIS expression with the differentiation of thyroid cancers has been studied, but the results are not consistent. It has been reported that the down-regulation of NIS is closely related to the dedifferentiation state of ATC 5, 6, resulting in the declined ability of iodine intake and therefore the resistance to radioiodine in ATC cells 3, 7. On the other hand, NIS expression is found to be up-regulated in thyroid cancer, but mainly located in the cytoplasm rather than the plasma membrane and, therefore, loses the ability to intake iodine 8, 9. Furthermore, NIS mRNA is substantially expressed in ATC and its transcriptional activity can be modulated by epigenetic modulators such as the members of Let7 miRNA family after deacetylase inhibitors treatment 10-12. It would be of clinical value to address the above issues by analysing the state of NIS expression and especially its intracellular distribution pattern(s) in ATC cells under growth inhibition and or redifferentiation.

PTEN is known as a tumor suppressor gene, which acts as an antagonist of PI3K/Akt/mTOR signaling pathway to regulate cell proliferation and gene expression including NIS 5, 13. For instance, when rat thyroid follicular cell line FRTL-5 and human papillary thyroid cancer cell line BHP2-7 were treated by PI3K signaling pathway inhibitor, their NIS levels were elevated, acompanied with improved iodine uptake 5. In addition, knockout of PTEN inhibited the activity of UDPN-acetylglucosamine-dolichyl-phosphate N-acetylglucosamine-phosphotransferase (DPAGT1) and the glycosylation of NIS protein, resulting in the increased NIS intracellular distribution 13. Nevertheless, the direct interaction between PTEN and NIS remains unclear. It has been found that PTEN expression is often down regulated in thyroid cancer due to DNA methylation, and its methylation level is positively related to the transformation of benign thyroid disease to undifferentiated thyroid cancer 14-16. It would be reasonable to consider that promotion of PTEN expression may increase NIS production and therefore enhance radioiodine sensitivity of ATC cells.

Retinoic acid has been widely used in the treatment of tumor differentiation, but its anti-ATC efficacy is still in dispute. Kogai T. and Brent G. A. reported that retinoic acid could improve the therapeutic effect of radioiodine on refractory thyroid cancer by inducing NIS expression 17, while the same treatment failed to achieve the above results by other groups 18, 19. According to our recent findings, retinoic acid, promoted proliferation of ATC cells, because of the methylation-silenced CRABP2 expression 20. Resveratrol, a natural polyphenol compound, has multiple biological functions such as induction of differentiation with E-cadherin membranous localization 21-23, suppression of cancer growth 24-26 and erase of DNA methylation 27. These points have also been confirmed in ATC *in vitro* experiment system 20, 28, 29. More importantly, Res in the anticancer doses has little toxic effects on normal tissues or cells, suggesting its potential values in better management of ATCs 30, 31. The current study aims to elucidate the statuses of PTEN and NIS expression and distribution patterns in Res-suppressed ATC cells.

## Material and Methods

### Clinical samples and immunohistochemistry

The paraffin-embedded ATC specimens were kindly provided by Department of Clinical Pathology, Guangdong Provincial People's Hospital, Guangzhou, China. All experiments were conducted with the approval of the Medical Ethics Committee of Guangdong Provincial People's Hospital. And all participating patients signed the informed consent before sample collection. The sections were dewaxed, rehydrated, and antigen retrieval (microwaved in 10 mM citrate buffer, pH 6.0 for 20 min), followed by blocking buffer. Further, the sections were incubated with primary antibodies (rabbit anti-NIS polyclonal antibody, 1:250, Bioss, bs-0448R, Beijing, China; rabbit anti-PTEN polyclonal antibody, 1:250, Wanleibio, WL01901, Shenyang, China) overnight at 4°C, and the following day, the sections were washed 3 times with PBS and incubated for 30 minutes with a secondary antibody, and then incubated with horseradish labeled streptomycin-antibiotin working solution for 15 minutes at 37°C. Finally, the color reaction was stained with 3,3'-diaminobenzidine tetrahydrochloride (DAB), and counterstained with hematoxylin for 30 seconds. Pictures were taken by a light microscope (Olympus).

### Cell culture and drug treatment

The ATC cell lines THJ-16T, 21T were provided by Dr. Liu Q. (Institute of Cancer Stem Cell, Dalian Medical University, as the general gifts of Mayo Foundation for Medical Education and Research). The human thyroid epithelial cell line Nthy-ori 3-1 was cited as the control to elucidate the effect(s) of resveratrol on the normal thyroid cells 32. The above three cell lines were cultured in RPMI 1640 (Gibco, Thermo Fisher Scientific, Suzhou, China) supplemented with 10% fetal bovine serum (Gibco Life Science, Grand Island, NY, USA) for THJ-21T and Nthy-ori 3-1 cells, and with 5% fetal bovine serum for THJ-16T, 100 IU/ml penicillin, and 100 μg/ml streptomycin in a humidified atmosphere of 5% CO_2_ in air at 37°C. Res (Sigma-Aldrich, USA) was dissolved in dimethyl sulfoxide (DMSO; Sigma-Aldrich, USA) to 100 mM as stock solution. THJ-16T, THJ-21T and Nthy-ori 3-1 cells were treated with 100 μM Res as previously reported 20, 33.

### Analyses of cell proliferation and apoptosis

Hematoxylin-Eosin (H/E) staining was performed on cell-bearing coverslips to observe the morphological changes of Res-treated THJ-16T, THJ-21T and Nthy-ori 3-1 cells by the method described elsewhere 34. 3-[4,5-Dimethylthiazol-2-yl]-2,5-diphenyl-tetrazolium bromide (MTT) assay, EdU staining were performed to elucidate proliferative activity and trypan blue cell discrimination assay for cell death rates. Deoxynucleotidyl transferase-mediated dUTP-biotin nick and labeling assay (TUNEL, Beyotime Biotechnology, C1086, Shanghai, China) was used to analyze apoptotic cell death by the methods described previously 33. The cell images were collected under a positive fluorescence microscope (Zeiss, Ax10 Axio, Germany).

### Reverse transcription-polymerase chain reaction (RT-PCR)

The RNA (OMEGA, R6934-01, USA) was isolated from THJ-16T and THJ-21T cells without and with resveratrol treatment for 48 h. Nanodrop (Thermo Scientific) was used to evaluate the quality and concentration of RNA extracted. Reverse transcription was performed using Takara PrimeScript^TM^ RT reagent kit (Takara Biotechnology Co., Ltd., RR037A, Japan). Briefly, 0.5 μg of each RNA sample was added to 10 μl of RT reaction mixture containing 2 μl of 5× PrimeScript buffer, 0.5 μl of PrimeScript RT Enzyme Mix Ⅰ, 0.5 μl of oligo dT-adaptor primer, 0.5 μl of random 6 mers and RNase-free distilled H_2_O up to 10 μl. The reaction was carried out at 37°C for 15 min, at 85°C for 5 sec. The synthesized cDNA was used as a template for the PCR reaction using Premix Taq^TM^ (Takara Biotechnology Co., Ltd., RR902A, Japan) and primers for target genes are listed in Table 1. Furthermore, RT-PCR was carried out by Bio-Rad T100 thermal cycler (Bio-Rad, Richmond, CA). The PCR condition was performed as follows: the samples were subjected to 30 cycles at 98°C for 10 s, 55°C for 30 s, 72°C for 60 s, the samples were stored at 4°C. Agarose gels (1.0%) containing ethidium bromide (0.5 mg/mL) were prepared for the separation of the PCR products, and BIO-RAD Gel Doc^TM^ XR^+^ with Image Lab^TM^ Software (BIO-RAD, USA) was used to visualize and photograph the samples. mRNA levels were normalized to levels of GAPDH.

### Protein preparation and Western blotting

Cells were treated with Res for 48 h, and then washed with ice-cold phosphate-buffered saline (PBS) three times and then lysed by RIPA buffer containing protease and phosphatase inhibitors. The protein concentrations were detected by bicinchoninic acid (BCA) protein quantification kit (Beyotime, P0012, Shanghai, China). Western bloting was performed by the method described elsewhere 31. Briefly, proteins were separated by 10% SDS-PAGE and transferred to polyvinylidene difluoride membrane. The membrane was blocked by 5% skimmed milk in Tris-buffered saline (TBS-T) for 3 hours, followed by incubated with the primary antibody (rabbit anti-human NIS polyclonal antibody, 1:600; rabbit anti-human PTEN polyclonal antibody, 1:1000; rabbit anti-human E-cadherin polyclonal antibody, 1:1000, Proteintech, 20874-1-AP, Chicago, USA; rabbit anti-human GAPDH polyclonal antibody, 1:2000, Wanleibio, WL01547, Shenyang, China) overnight at 4°C. The next day, the primary antibody was discarded, and then the membrane was washed three times by TBST, followed by 1h incubation with horseradish peroxidase (HRP)-conjugated anti-rabbit IgG. The bands were visualized by the ECL system (Amersham Imager600, GE Healthcare Life Sciences, USA). The labeling signal was removed with a stripping buffer, and the membrane was incubated with another primary antibody until all the parameters were examined.

### Immunocytochemical and double immunofluorescent staining

Immunocytochemical staining/ICC was performed on the cell-bearing coverslips obtained from each of the experimental groups by the method described elsewhere 31. The antibodies used were: NIS (Bioss, bs-0448R, Beijing, China; 1:500), PTEN (Wanleibio, WL01901, Shenyang, China; 1:250), E-cadherin (Proteintech, 20874-1-AP, Chicago, USA; 1:500). The color reaction was performed by using 3,30-diaminobenzidine tetrahydrochloride (DAB). For double immunofluorescent staining/IF, after washed with PBS 3 times and then blocked by normal goat serum for 30 minutes at 37°C, the cell-bearing coverslips were co-incubated with rabbit anti-NIS (1:250) and mouse anti-PTEN (Bioss, bsm-33320M, Beijing, China; 1:250) overnight at 4°C in a humid chamber. Then, the coverslips were co-incubated with coralite488-conjugated affinipure goat anti-rabbit IgG (1:500, Proteintech, SA00013-2, Chicago, USA) and coralite594-conjugated goat anti-mouse IgG (1:500, Proteintech, SA00013-3, Chicago, USA) at 37°C for 60 minutes in the dark. Cell nucleus were stained with Hoechst, sealed with fluorescence mounting medium, and observed and imaged under a positive fluorescence microscope (Zeiss, Ax10 Axio, Germany).

### Statistical analyses

Each experiment was conducted for three times, and the data obtained were analyzed together. The results of cell proliferation and cell apoptosis assay were evaluated with the independent-samples t-test. The band of RT-PCR and Western Blotting were analyzed by Image J. The bar graphs present the mean ± standard deviation (SD) of separate experiments. * indicates *p* < 0.05, and ** indicates *p* < 0.01, which were all considered significance.

## Results

### Different effects of resveratrol on ATC cells and their normal counterpart

**Figure 1:**
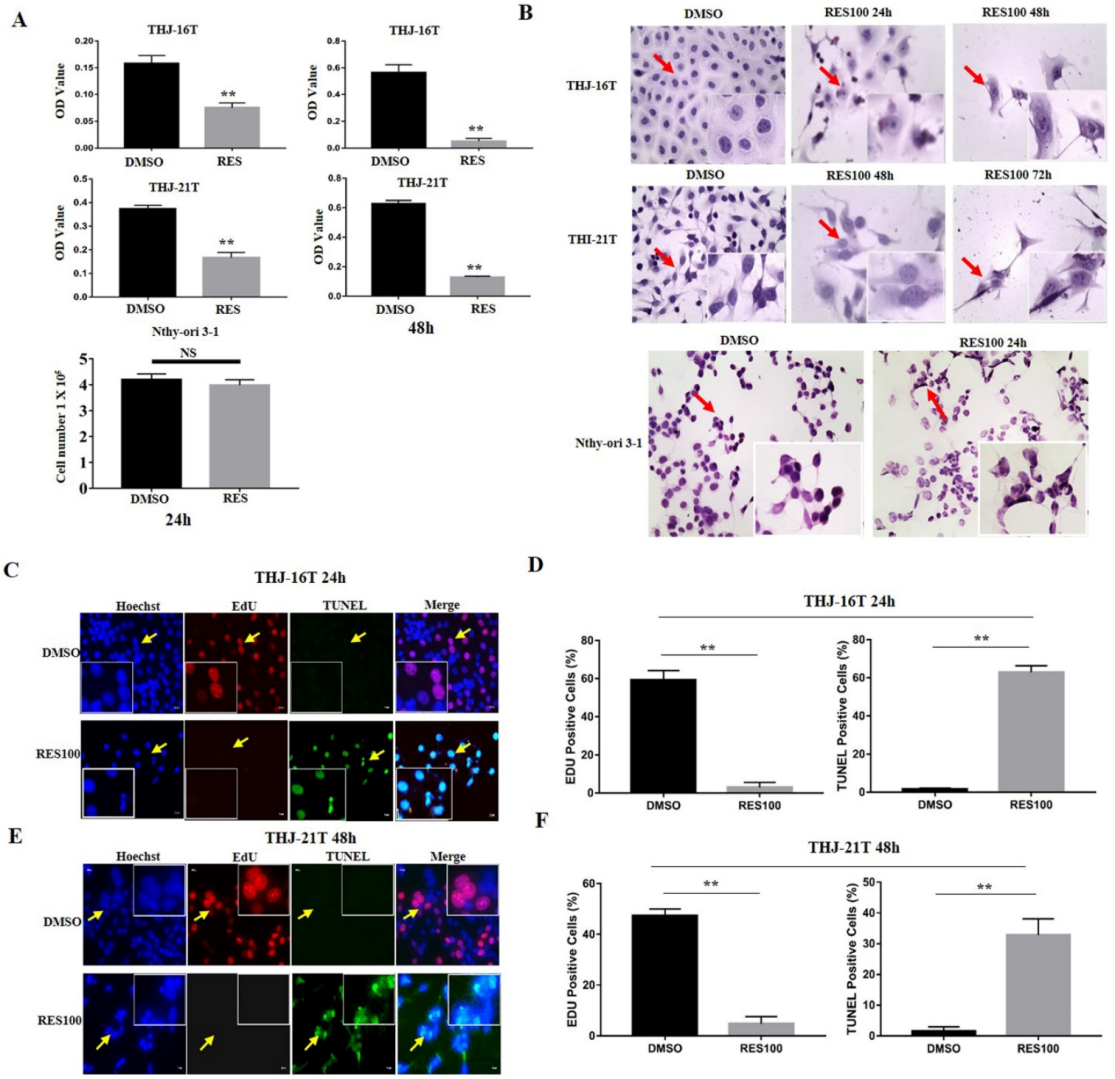
** Effects of resveratrol on proliferation and apoptosis of THJ-16T, THJ-21T and Nthy-ori 3-1 cells.** (**A**) MTT proliferative assay and trypan blue cell discrimination assay; (**B**) H/E (x 40) morphological staining; (**C-F**) EdU and TUNEL staining (x 40). ** indicates *p* < 0.01 was considered significance; NS, without statistical significance (*p* > 0.05); the error bars, the mean ± standard deviation (SD). The arrow indicates the enlarged area of the insert image (x 80).

MTT cell proliferation assay demonstrates that after Res treatment for 24 h and 48 h, the optical density (OD) values of THJ-16T and THJ-21T cells decreased obviously compared with those of 0.1% DMSO treated cells in time-related fashion (*p* < 0.01; Figure 1A). H/E morphological staining reveals that after 100 µM Res treatment, THJ-16T and THJ-21T cells show extensive cell death and the morphology of cells changed obviously (Figure 1B). EdU positive cells were significantly decreased (2.98% vs 59.16% of THJ-16T, *p* < 0.01 and 4.74% vs 47.35% of THJ-21T, *p* < 0.01) and TUNEL positive cells were increased in Res treatment group compared with those cells cultured in 0.1% DMSO-containing medium (1.52% vs 62.86% of THJ-16T, *p* < 0.01 and 1.62% vs 39.58% of THJ-21T, *p* < 0.01; Figure 1C-1F). In contrast, Res-treated Nthy-ori 3-1 cells showed a 5.1% decrease in comparison with DMSO-treated group (Figure 1A; *p* > 0.05) without distinct morphological change (Figure 1B).

### Differential NIS and PTEN expression in ATC and normal thyroid cells

**Figure 2:**
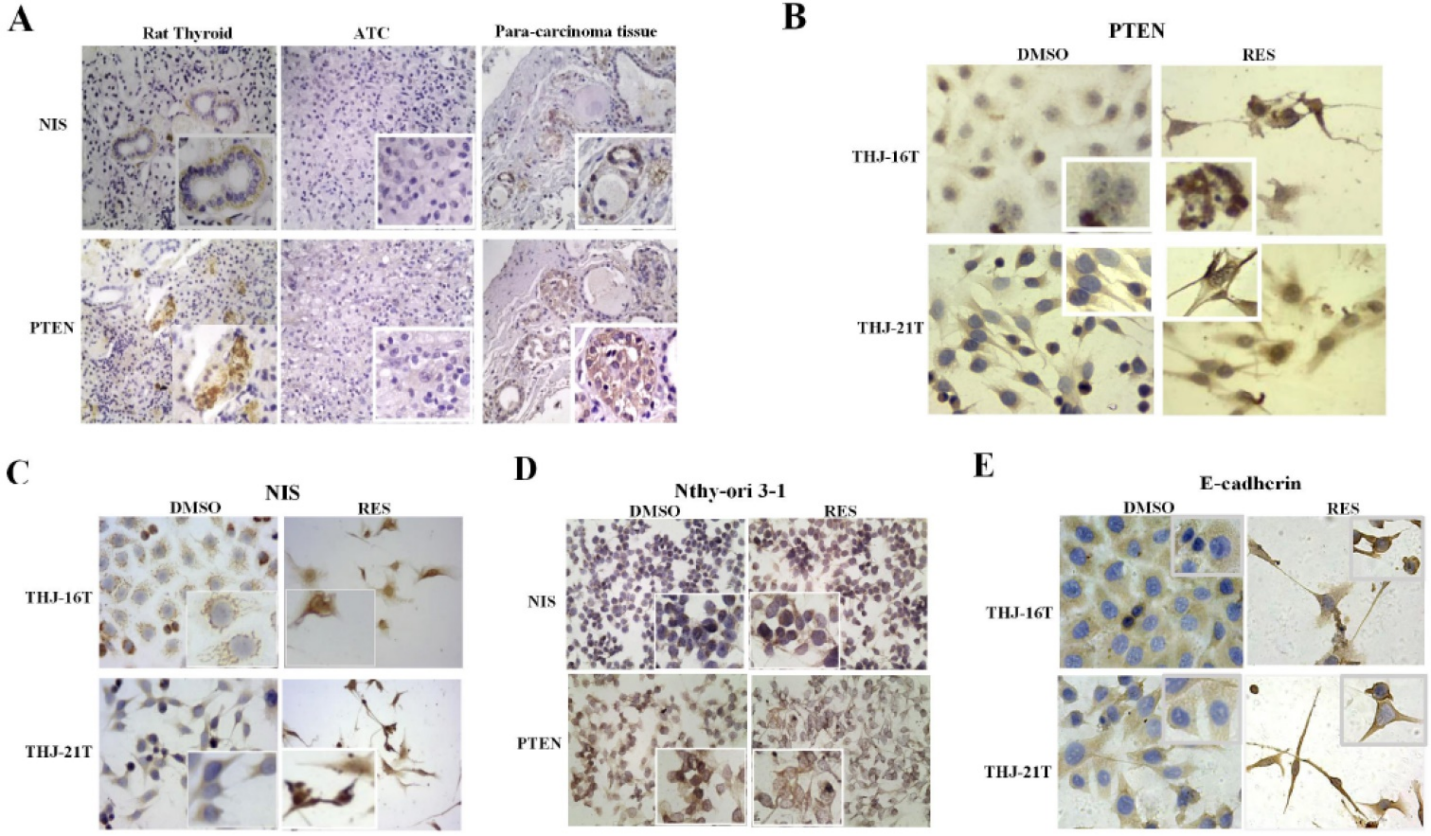
** Intracellular distribution of NIS, PTEN and E-cadherin proteins in ATC and normal thyroid cells without and with resveratrol treatment.** (**A**) Immunohistochemistry/IHC (x 40) in ATC and para-carcinoma tissues and rat normal thyroid tissues; (**B-E**) Immunocytochemistry/ICC (x 40) in THJ-16T, THJ-21T and Nthy-ori 3-1 cells. The images in higher magnification (x 80) were shown in the insets.

Because the expression level of NIS and PTEN is known to be closely related to the differentiation degree of thyroid cancer, the expression of these two proteins in ATC cells, normal thyroid cell line, the normal rat thyroid tissues and noncancerous tumor surrounding thyroid tissues were examined. The results of immonuhistochemical staining showed that NIS and PTEN were positive in normal thyroid cell line, normal thyroid tissue and noncancerous thyroid body. NIS was mainly distributed in basement membrane of normal thyroid cell line and normal thyroid follicular cells, and PTEN mainly in nucleus (Figure 2A and 2D). On the contrary, the expression of PTEN and NIS in ATC cells were significantly decreased in the form of negative or weakly positive staining in the cytoplasm (Figure 2B and 2C).

### Distinct NIS nuclear translocation in resveratrol-treated ATC cells

**Figure 3:**
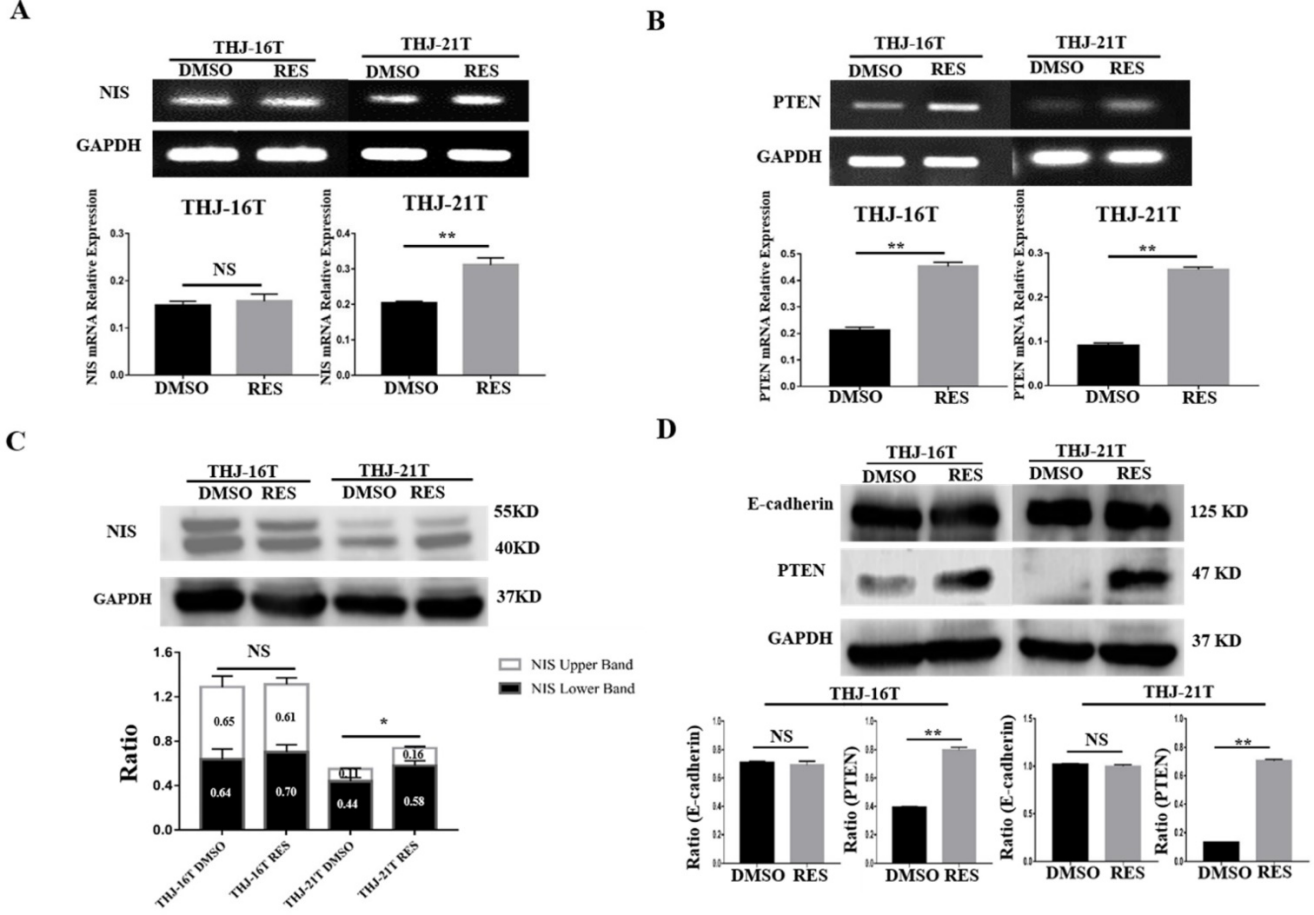
** Differential expression of NIS, PTEN and E-cadherin in THJ-16T and THJ-21T cells without and with 100 µM Res treatment.** RT-PCR (**A and B**) and Western blotting (**C and D**) were performed on the two cell lines without and with 100 µM Res treatment. Ratio, the ratio between the levels of the target molecules and that of GAPDH. *, with statistical significance (*p* < 0.05); **, with statistical significance (*p* < 0.01); NS, without statistical significance (*p* > 0.05); the error bars, the mean ± standard deviation (SD).

RT-PCR and Western blotting were conducted to analyze NIS expression in THJ-16T and THJ-21T cells before and after Res treatment. Previous studies have shown that the membrane NIS protein bands have different positions, some at 86 KD 35, and some at 50 KD 36, while the bands detected in this study are between 40-55 KD, which are double bands, consistent with Byeong-Cheol Ahn's report 37. Compared with DMSO control group, the expression of NIS in THJ-21T cells was significantly increased after Res treatment. However, no obvious change of NIS levels was found in THJ-16T cells (Figure 3A and 3C). In addition to the expression level, the intracellular distribution patterns are another important factor affecting the function of NIS proteins. Immunocytochemistry was used to observe NIS distribution pattern in the two Res treated ATC cell lines and normal thyroid cell line. The results showed the increased NIS immunolabeling, especially in the nuclei of the former two rather than the normal one (Figure 2C and Figure 2D).

### Resveratrol promoted E-cadherin membrane localization

The close correlation of E-cadherin expression and, especially, membranous distribution with ATC cell re-differentiation has been ascertained by our previous investigation 20. We therefore cited membranous E-cadherin as a re-differentiation biomarker of ATC cells in current study. The expression and distribution of E-cadherin in THJ-16T and THJ-21T before and after resveratrol treatment were analysed. The results of Western blotting showed no significant change of E-cadherin expression in the two cell lines before and after resveratrol treatment (*p* > 0.05; Figure 3D). ICC staining showed that E-cadherin was mainly distributed in the cytoplasm before resveratrol treatment, which appeared on the out membrane of resveratrol-treated THJ-16T and THJ-21T (Figure 2E).

### Resveratrol upregulated PTEN expression in ATC cells

**Figure 4:**
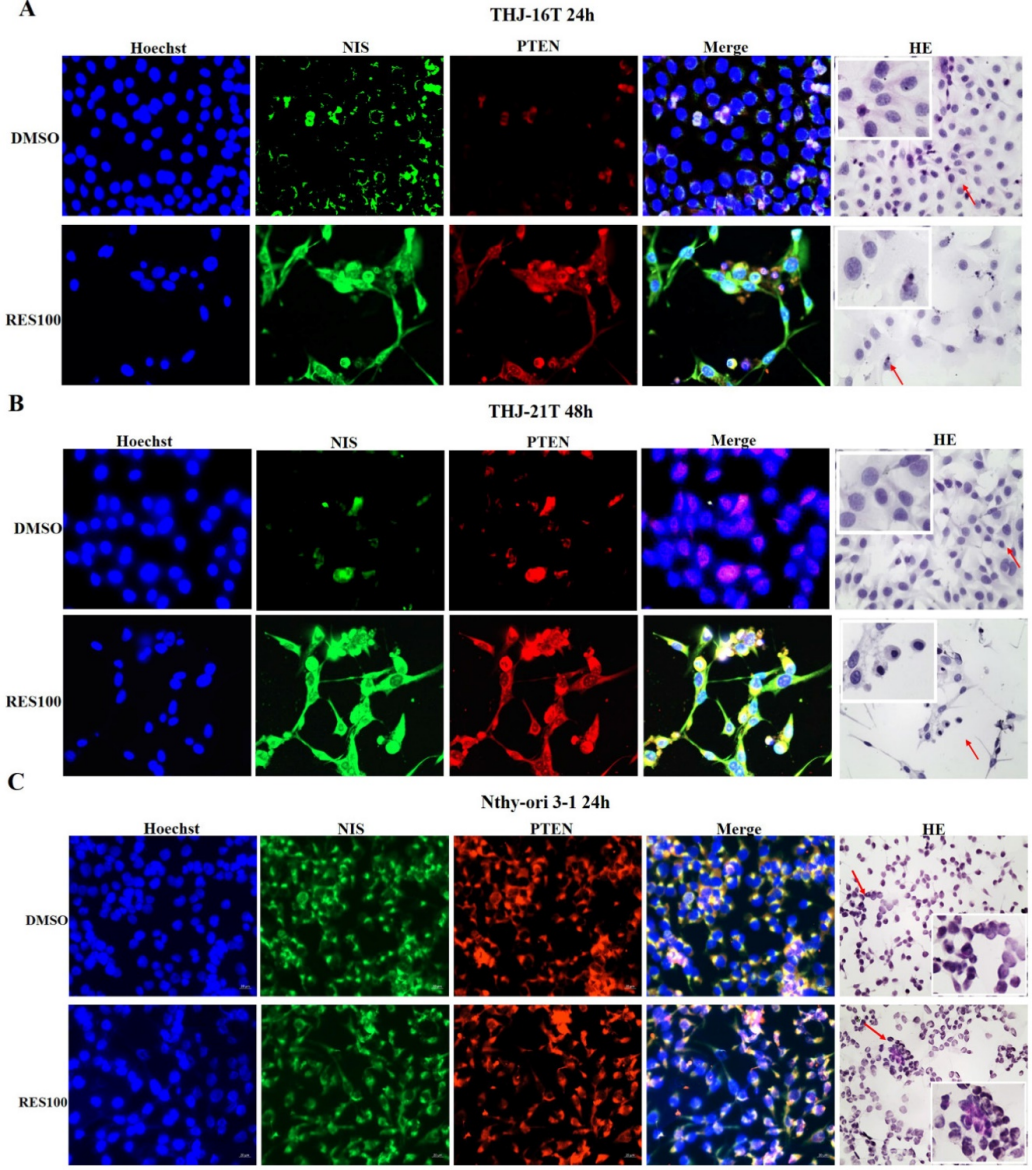
** Immunofluorescent illustration of NIS and PTEN intracellular distribution patterns in ATC and normal thyroid cells before and after resveratrol treatment.** NIS and PTEN oriented double immunofluorescent labeling (x 40) was performed on THJ-16T (**A**), THJ-21T (**B**) and Nthy-ori 3-1 cells (**C**), paralelled with H/E morphological staining (x40). Arrows indicate the portions with higher magnification (x 80) in the insets.

RT-PCR and Western blot analyses revealed that the mRNA and protein levels of PTEN were 2.06 and 2.03 folds increased in THJ-16T cells and 2.90 and 5.36 folds increased in THJ-21T cells, respectively (Figure 3B and 3D). PTEN protein was mainly distributed in the cytoplasm of THJ-16T, THJ-21T cells before Res treatment. After Res treatment, distinct PTEN nuclear translocation was observed in both ATC cell lines (Figure 2B). PTEN and NIS oriented double immunofluorescent labeling showed obvious concurrent nuclear translocation of these two proteins in the two ATC cell lines (Figure 4A and 4B). The PTEN expression levels and intracellular distribution pattern of Nthy-ori 3-1 cells remained similar irrespective to resveratrol treatment (Figure 2D and Figure 4C).

## Discussion

Several approaches have been employed in the treatment of ATCs, including surgery, radiotherapy, chemotherapy, molecular targeted therapy and their combination, but the therapeutic outcome is very poor 2, 38. ^131^I internal irradiation is commonly used in post-operative adjuvant therapy for differentiated thyroid cancer, its effectiveness depends on the level of NIS expression because amount of iodine intake is largely mediated by this protein 39. It has been found that NIS, irrespective to its level, is usually distributed in the cytoplasm, leading to the decrease of iodine uptake 3, 7-9. This point is further confirmed in this study that the NIS level of ATC tissue and cell line is significantly lower than that of the normal rat thyroid glands as well as human noncancerous ATC surrounding tissue, and NIS proteins are distributed in the cytoplasm rather than on the membrance. This finding suggests that the down-regulation of NIS expression and its absence on the outer membrane are common molecular biological alterations of ATCs, which may be one of the results of dedifferentiation. Res is known to promote the differentiation of ATC cells 28. We therefore supposed that Res might inhibit the proliferation of ATC cells and meanwhile make NIS re-localized on the cell membrane to restore the iodine uptake function of the treated cells. If this speculation is confirmed, Res can replace retinoic acid as a new ATC inhibitor and iodine radiosensitizer.

Given the evidence of the close correlation between NIS downregulation and the dedifferentiation of ATCs and the ability of Res to promote ATC cell differentiation, it would be possible that NIS expression will be elevated and NIS proteins may re-appear on the membrane of Res-treated ATC cells as what is happened to E-cadherin 20. To address these issues, the expression and distribution of NIS in two ATC cell lines and human normal thyroid cell line Nthy-ori 3-1 before and after Res treatment were analysed. It was found that NIS expression was up-regulated in THJ-21T cells after Res treatment, while it remained almost unchanged in THJ-16T cells. More interestingly, unlike the situation of E-cadherin, NIS did not localize on the cell surface but transferred into the nuclei of Res-treated THJ-16T and THJ-21T cells. In contrast, Res exerts little influence in proliferation and NIS intracellular distribution of Nthy-ori 3-1 cells, indicating the ATC-selective effects and nontoxic feature for normal thyroid cells of this polyphenol compound 30. This phenomenon suggests that even if ATC cells are differentiated and NIS expression is up-regulated, it is difficult to improve their iodine uptake. The nuclear translocation of NIS proteins is a new finding of current study 3, 13. We are exploring the underlying reason as well as the potential biological implication of this event as the next step of our investigation.

It has been recognized that PI3K/Akt/mTOR signaling pathway is the main regulation machinary of NIS expression 5, 40. PTEN, a tumor suppressor gene, acts as the upstream regulator of PI3K/Akt/mTOR signaling pathway, which inhibits the activation of PI3K/Akt/mTOR signaling via suppressing the transformation of PIP2 into PIP3 41. The absence of PTEN may enhance PI3K/Akt/mTOR signaling activity, resulting in NIS deglycosylation, and cytosolic NIS aggregation 13, 40. According to the literature, PTEN is expressed in normal tissues but remarkable reduced in their malignant counterparts, which plays a role in promoting tumor cell proliferation 42, 43. Therefore, the presence or absence of PTEN expression has been regarded as a prognostic factor of some cancers including ATCs 44, 45. It was found in current study that compared with the tumor surrounding tissues and rat normal thyroid tissues, PTEN expression in ATC tissues was downregulated. After Res treatment, PTEN expression increased significantly, and with obvious nuclear translocation. DNA methylation is known as the main cause of PTEN reduction 14-16. Our previous study found that Res upregulated CRABP2 expression by erasing its promoter methylation, and therefore reversed retinoic acid resistance of the two ATC cell lines used in this study 27. In this context, it is reasonable to consider that Res may upregulate PTEN expression in the same manner.

It has been known that PTEN, as a transcriptional factor, needs to enter the nucleus to conduct its biological functions such as maintain chromosome stability, DNA repair and cell cycle arrest 46-49. Interestingly, both NIS and PTEN proteins have a putative density-95/discs large/zona clusters-1 (PDZ) structure in the secondary structure, which enables them to combine with each other through the PDZ-PDZ domain and then translocate into the nucleus 4, 50. In other words, NIS protein can interact with PTEN to form a complex and increased PTEN production permitts NIS proteins have more chance to be translocated into the nucleus as demonstrated in our PTEN and NIS double immunofluorescent labeling. Although the biological significance of PTEN/NIS nuclear co-translocation remains to be elucidated, this phenomenon may indicate a better therapeutic outcome and favorable prognosis of ATCs.

## Figures and Tables

**Table 1:** Primer sequences for RT-PCR

Gene	5'-3' forward primer	5'-3' reverse primer
NIS	CCA CCG GAA TTA TCT GCA CCT	ACG ACC TGG AAC ACA TCA GTC
PTEN	TTT GAA GAC CAT AAC CCA CCA C	ATT ACA CCA GTT CGT CCC TTT C
GAPDH	CTC AAC GAC CAC TTT GTC AAG CTC	CTC TCT TCC TCT TGT GCT CTT GCT
